# *GRIN1* Mutations in Early-Onset Epileptic Encephalopathy

**DOI:** 10.15844/pedneurbriefs-29-6-3

**Published:** 2015-06

**Authors:** Wenjuan Chen, Hongjie Yuan

**Affiliations:** 1 Department of Pharmacology, Emory University, Atlanta, GA

**Keywords:** NDMA receptors, GluN1, Epilepsy

## Abstract

Investigators from Yokohama City University and other medical centers in Israel and Japan reported mutations on N-methyl-D-aspartate (NMDA) receptors subunit *GRIN1* (GluN1) identified in patients with nonsyndromic intellectual disability and early-onset epileptic encephalopathy.

Investigators from Yokohama City University and other medical centers in Israel and Japan reported mutations on N-methyl-D-aspartate (NMDA) receptors subunit *GRIN1* (GluN1) identified in patients with nonsyndromic intellectual disability and early-onset epileptic encephalopathy. Next generation sequencing analysis of 88 unsolved epileptic encephalopathies revealed 4 patients with 4 *de novo* missense *GRIN1* mutations. In these 4 patients, initial symptoms appeared within 3 months of birth, including hyperkinetic movements (2/4), and seizures (2/4). All developed different types of seizures during first year of life including spasms, myoclonic and focal. EEG showed nonspecific focal and diffuse epileptiform abnormality, and never showed suppression-burst or hypsarhythmia during infancy. Involuntary movements, severe developmental delay, and intellectual disability occurred in all four patients. Brain MRI images of all four patients showed cerebral cortex atrophy, and/or ventriculomegaly, and/or thin corpus callosum. Three mutations were located in the transmembrane domain, and 1 in the extracellular loop near transmembrane helix 1; all were predicted to impair the function of the NMDA receptor. [1]

COMMENTARY. Dramatic advances in next-generation sequencing technologies have led to a rapid increase in the amount of exome sequencing data, which has advanced our understanding of the genetic basis of neurologic diseases, including epilepsy. In recent years, a surprising number of missense mutations and deletions/truncations (>140) have been identified in NMDA receptors through whole exome sequencing [2]. These mutations are scattered across all domains in NMDA receptor subunits, including the *GRIN1* gene, which encodes the GluN1 subunit. Moreover, these mutations appear associated with multiple neuropathological conditions, for which epilepsy/seizures comprise the largest group [2]. *GRIN2A*/GluN2A subunit constitutes a locus for mutations in a subset of patients with early-onset epilepsy [2]. This report suggests that the *GRIN1*/GluN1 subunit may be another locus for early-onset seizures.

Despite the identification of new NMDA receptor mutations, there are still only minimal functional data available for a handful of *de novo* mutations [2]. One noteworthy example is GluN2A(L812M), which was identified in a patient with intractable seizures and early-onset epileptic encephalopathy [3]. Functional studies of this mutation suggested that a profound increase in receptor function likely contributes to the patient's phenotype. Evaluation of the potency of FDA-approved drugs that can block mutant NMDA receptors raised the possibility of personalized therapies. When the NMDA receptor channel blocker memantine was introduced into the treatment regimen for this patient, it significantly reduced seizure frequency [4]. While this effect remains to be replicated in other patients and rigorously tested in clinical trials, the example emphasizes the need and potential promise of functional analysis of the rapidly expanding list of NMDA receptor mutations revealed by gene sequencing programs for patients. Locations of the reported *GRIN1* mutants were in pre-M1 helix (D552E), M3 transmembrane domain (M641I and N650K), and M4 transmembrane domain (G815R), all which are considered as important elements/domains that are likely to influence NMDA receptor gating. We expect that future studies of the effects of these *GRIN1* mutations on NMDA receptor functions will be useful not only for clarifying molecular mechanism underlying these patients’ phenotype, but also for exploring the potential targeted therapies.
